# Management of allergic rhinitis with leukotriene receptor antagonists versus selective H1-antihistamines: a meta-analysis of current evidence

**DOI:** 10.1186/s13223-021-00564-z

**Published:** 2021-06-29

**Authors:** Yan Feng, Ya-Ping Meng, Ying-Ying Dong, Chang-Yu Qiu, Lei Cheng

**Affiliations:** 1 Department of Otolaryngology-Head and Neck Surgery, The First Hospital, Shanxi Medical University, Taiyuan, China; 2 Shanxi Key Laboratory of Otorhinolaryngology-Head and Neck Cancer, Taiyuan, China; 3 Henan Vocational College of Applied Technology, Zhengzhou, China; 4 Department of Otorhinolaryngology & Clinical Allergy Center, The First Affiliated Hospital, Nanjing Medical University, 300 Guangzhou Road, Nanjing, 210029 China; 5 International Centre for Allergy Research, Nanjing Medical University, Nanjing, China

**Keywords:** Allergic rhinitis, Leukotriene receptor antagonists, H1-antihistamines, Randomized controlled trials, Meta-analysis

## Abstract

**Background:**

Inconsistencies remain regarding the effectiveness and safety of leukotriene receptor antagonists (LTRAs) and selective H1-antihistamines (SAHs) for allergic rhinitis (AR). A meta-analysis of randomized controlled trials (RCTs) was conducted to compare the medications.

**Methods:**

Relevant head-to-head comparative RCTs were retrieved by searching the PubMed, Embase, and Cochrane’s Library databases from inception to April 20, 2020. A random-effects model was applied to pool the results. Subgroup analyses were performed for seasonal and perennial AR.

**Results:**

Fourteen RCTs comprising 4458 patients were included. LTRAs were inferior to SAHs in terms of the daytime nasal symptoms score (mean difference [MD]: 0.05, 95% confidence interval [CI] 0.02 to 0.08, *p* = 0.003, *I*^2^ = 89%) and daytime eye symptoms score (MD: 0.05, 95% CI 0.01 to 0.08, *p* = 0.009, *I*^2^ = 89%), but were superior in terms of the nighttime symptoms score (MD: − 0.04, 95% CI − 0.06 to − 0.02, *p* < 0.001, *I*^2^ = 85%). The effects of the two treatments on the composite symptom score (MD: 0.02, 95% CI − 0.02 to 0.05, *p* = 0.30, *I*^2^ = 91%) and rhinoconjunctivitis quality-of-life questionnaire (RQLQ) (MD: 0.01, 95% CI − 0.05 to 0.07, *p* = 0.71, *I*^2^ = 99%) were similar. Incidences of adverse events were comparable (odds ratio [OR]: 0.97, 95% CI 0.75 to 1.25, *p* = 0.98, *I*^2^ = 0%). These results were mainly obtained from studies on seasonal AR. No significant publication bias was detected.

**Conclusions:**

Although both treatments are safe and effective in improving the quality of life (QoL) in AR patients, LTRAs are more effective in improving nighttime symptoms but less effective in improving daytime nasal symptoms compared to SAHs.

## Background

Allergic rhinitis (AR) is a common allergic disease caused by immunoglobulin E (IgE)-associated inflammation of the nasal membranes as a result of exposure to allergens [1, 2]. AR can be categorized as seasonal or perennial according to the persistence of the symptoms. Patients with AR are affected by nasal and eye symptoms, which interrupt their daily lives and sleep schedule, leading to impaired QoL [3]. The primary treatments for AR are allergen avoidance, pharmacotherapy, and immunotherapy [4, 5]. Among the oral medications available to relieve the symptoms of AR, leukotriene receptor antagonists (LTRAs) and selective H1-antihistamines (SAHs) are commonly prescribed [6]. By blocking cysteinyl leukotriene-activated inflammation in the nasal lavage fluids and airways, LTRAs effectively attenuate nasal obstruction and rhinorrhea [7]. SAHs selectively inhibit histamine 1 receptor (H1R)-mediated vasopermeability and vasodilatation and are widely utilized for relieving rhinorrhea and congestion in AR [8]. However, previous randomized controlled trials (RCTs) comparing the efficacy and safety of LTRAs and SAHs for patients with AR yielded inconsistent results [9–22]. Consequently, the recommendations for LTRA and SAH use for AR patients vary in different international guidelines [23]. The 2015 US Clinical Practice Guidelines for Allergic Rhinitis recommend oral second-generation/less sedating antihistamines for patients with AR who have primary complaints of sneezing and itching, but do not recommend LTRAs as the primary therapy for patients with AR [24]. In contrast, the 2017 Japanese Guidelines for Allergic Rhinitis suggest that LTRAs may be comparable to SAHs for sneezing and rhinorrhea in patients with moderate or mild nasal blockage [25]. The recent 2018 Chinese Society of Allergy Guidelines for Diagnosis and Treatment of Allergic Rhinitis suggest that LTRAs and SAHs may have similar efficacy, but that LTRAs may be better suited for night-time symptoms [26]. In view of the discrepancies regarding the role of LTRAs and SAHs in the treatment of AR, we aimed to perform a meta-analysis of head-to-head RCTs to compare the effects of the two medications on the symptoms, QoL, and adverse events (AEs) in patients with AR.

## Methods

The PRISMA (Preferred Reporting Items for Systematic Reviews and Meta-Analyses) statement [27] and the Cochrane Handbook guidelines [28] were followed during the design and implementation of the study.

### Search strategy

PubMed, Embase, and the Cochrane Library (Cochrane Center Register of Controlled Trials) databases were systematically searched for relevant studies using the following combined strategy: (1) “leukotriene receptor antagonist” OR “LTRA” OR “montelukast” OR “zafirlukast” OR “pranlukast”; (2) “selective H1-antihistamine” OR “SAH” OR “cetirizine” OR “ebastine” OR “loratadine” OR “desloratadine” OR “acrivastine” OR “fexofenadine” OR “levocetirizine” OR “rupatadine”; (3) “allergic rhinitis”; and (4) “random” OR “randomized” OR “randomised” OR “randomly”. Only clinical studies published in English or Chinese were considered. The reference lists for related reviews and original articles were also searched to complement the results. The latest database search was conducted on April 20, 2020.

### Study selection

The inclusion criteria were: (1) peer-reviewed articles in English or Chinese; (2) designed as RCTs; (3) included patients with AR who were randomly allocated to receive LTRAs or SAHs with or without concomitant treatments; (4) with a treatment duration of at least 1 week; and (5) at least one of the following outcomes: daytime nasal symptoms score (DNSS), nighttime symptoms score (NSS), daytime eye symptoms score (DESS), composite symptoms score (CSS), RQLQ, and incidence of AEs. No restrictions were applied for the age of the patients or the blindness of the RCTs during the process of study inclusion. The DNSS includes four nasal symptoms (stuffy, runny, and itchy nose, and sneezing) and each symptom domain is scored from 0 to 3, with the highest score indicating the most serious symptoms. The DNSS is calculated as the sum of the scores (0–12) [29]. Similarly, the DESS includes four eye symptoms (teary, itchy, red, and puffy eyes) with a score of 0–3 for each domain and is calculated as the sum of the scores (0–12, 12 indicating the most serious symptoms) [29]. The NSS evaluates nighttime symptoms based on three factors (nasal congestion on awakening, difficulty going to sleep, nighttime awakenings) with a score of 0–3 for each domain and is calculated as the sum of the scores (0–9, 9 indicating the most serious symptoms) [29–31]. The CSS is defined as a post hoc composite score that captures the treatment effect over 24 h (mean of DNSS and NSS) [29–31]. The RQLQ assesses the QoL in AR patients via seven domains (sleep, non-nose and non-eye symptoms, practical problems, nasal symptoms, eye symptoms, activities, and emotions) via a total of 28 questions. The ratings for each of the questions range from 0–6 points and a sum of 168 points indicates the worst QoL [32]. The definitions of AEs were in accordance with the original articles. Reviews, preclinical studies, observational studies, and repeated reports were excluded.

### Data extraction and quality assessment

The study search, data extraction, and quality evaluation were performed independently by two of the authors and disagreements were resolved by consensus between them. We extracted data regarding the study information (first author, publication year, and study country), study design (blind or open-label, crossover or parallel design), patient information (seasonal or perennial AR, number of participants, mean age, gender, proportion of patients with asthma), treatment regimens (medications and doses of LTRA and SAH, and concomitant therapy), treatment duration, and outcomes reported. Quality evaluation was performed using the Cochrane’s Risk of Bias Tool [28] according to the following factors: (1) random sequence generation; (2) allocation concealment; (3) blinding of participants and personnel; (4) blinding of outcome assessors; (5) incomplete outcome data; (6) selective outcome reporting; and (7) other potential bias.

### Statistical analysis

The effects of LTRAs and SAHs on continuous outcomes, including DNSS, NSS, CSS, DESS, and RQLQ were summarized as differences in the changes in each outcome from the baseline between the groups. MD was used as the measure of the effect on the continuous outcome and the CIs were extracted. For categorized outcomes such as the incidence of AEs, OR and corresponding CIs were used. We used the Cochrane’s Q test to assess heterogeneity, and significant heterogeneity was suggested if *p* < 0.10 [33]. The *I*^2^ statistic was also calculated, and an *I*^2^ > 50% reflected significant heterogeneity. Pooled analyses were calculated using a random-effects model because this method incorporates the influence of potential heterogeneity and yields a more generalized result [28]. Sensitive analyses by excluding one dataset at a time were used to examine the stability of the findings. Subgroup analysis was also performed to evaluate the outcomes in patients with seasonal or perennial AR. Publication bias was evaluated by visual inspection of the funnel plots provided and by using Egger’s regression asymmetry test [34]. *p* values < 0.05 were considered statistically significant. RevMan (Version 5.1; Cochrane, Oxford, UK) and Stata software (Version 12.0; Stata, College Station, TX) were applied for statistical analyses.

## Results

### Search results

**Fig. 1:**
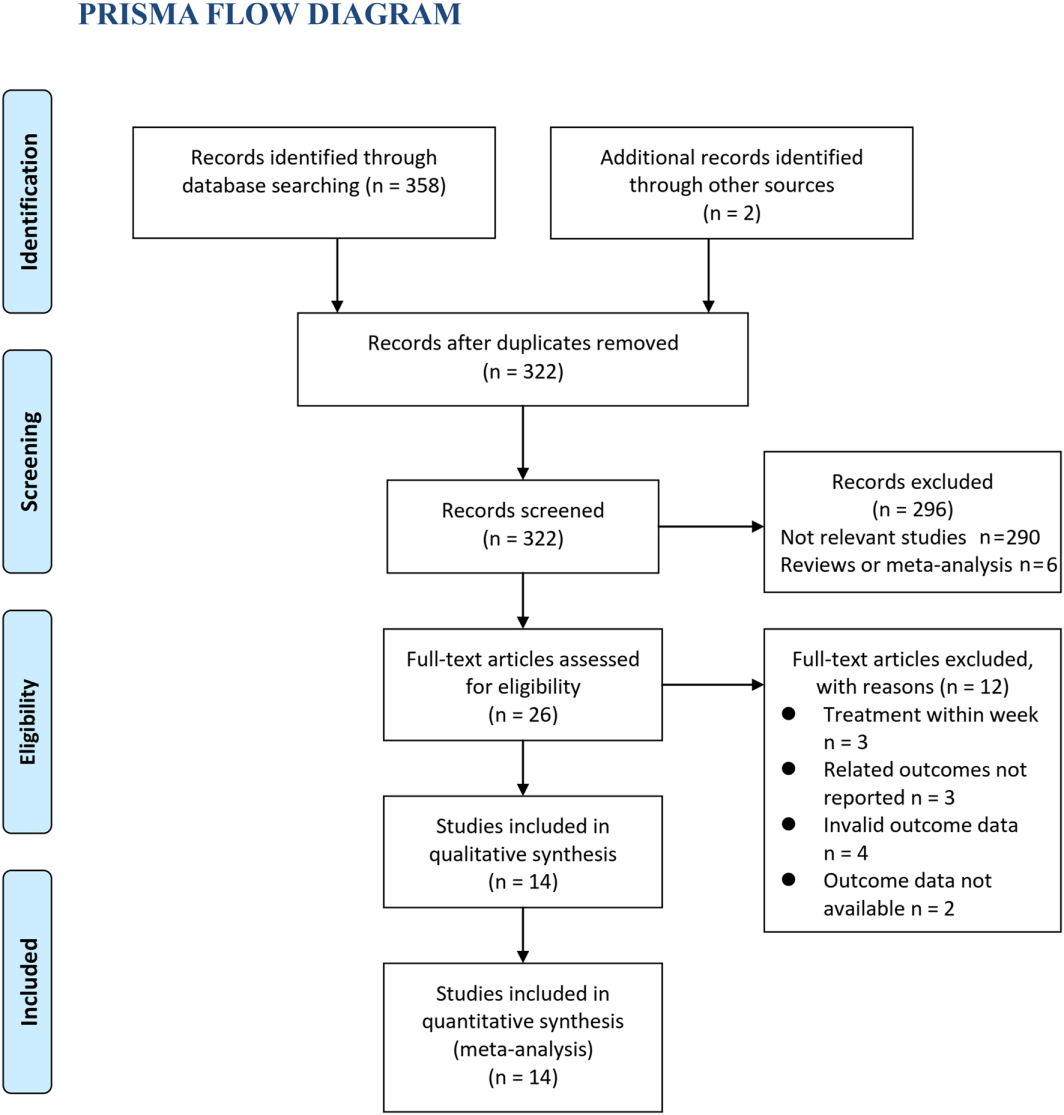
Flowchart of literature search

In summary, 322 articles were obtained through the database search after excluding duplicates. Among them, 296 articles were subsequently excluded primarily based on the titles and abstracts because the studies were not relevant. Among the 26 potentially relevant articles, 12 were further excluded after a full-text review due to the reasons shown in Fig. 1. Finally, 14 RCTs comprising 4458 patients with child and adult AR were included [9–22].

### Study characteristics

Table 1 shows the characteristics of the included studies. Overall, 14 RCTs [9–22] involving 4458 AR patients were included. One article included two RCTs [20], and another study [17] included two comparisons (montelukast 10 mg/d versus levocetirizine 5 mg/d, and montelukast 10 mg/d versus desloratadine 5 mg/d). These comparisons were included as independent datasets, resulting in a total of 16 datasets included in the meta-analysis. These studies were published between the years 2000 and 2017 and included AR patients from the United States, United Kingdom, Italy, Poland, and China. Eight of the studies included patients with seasonal AR [9–14, 20, 21], while six included perennial AR patients only [15–19, 22]. One study focused on pediatric patients (aged < 18 years) [15], two included only adult patients (aged ≥ 18 years) [16, 17], and the rest included both. For LTRA treatment, montelukast 10 mg/d was used in all but two studies in which montelukast 5 mg/d [15] and zafirlukast 40 mg/d [18] were used, respectively. For the SAHs, loratadine, fexofenadine, or desloratadine were used. Most of the included studies did not involve concomitant therapies for AR, although fluticasone propionate aqueous nasal spray was used in one study [14] and nasal mometasone was used for both groups in two studies [21, 22]. The treatment duration varied from 1 to 12 weeks.

**Table 1:** Characteristics of the included studies

Author (year)	Country	Design	Patients	Patient number	Mean age (year)	Male (%)	With asthma (%)	LTRAs	SAHs	Concurrent treatment for AR	Duration (weeks)	Outcomes
Meltzer 2000	The US	R, DB	SAR patients aged 15–75 y	187	33.6	44.4	31.1	Montelukast 10 mg/d	Loratadine 10 mg/d	None	2	DNSS, NSS, CSS, DESS, RQLQ
Nayak 2002	The US	R, DB	SAR patients aged 15–82 y	456	36.6	36.7	20.9	Montelukast 10 mg/d	Loratadine 10 mg/d	None	2	DNSS, NSS, CSS, DESS, RQLQ
Philip 2002	The US	R, DB	Non-smoking SAR patients aged 15–81 y	950	36.3	65.5	25.7	Montelukast 10 mg/d	Loratadine 10 mg/d	None	2	DNSS, NSS, CSS, DESS, RQLQ
van Adelsberg 2003a	The US	R, DB	Non-smoking SAR patients aged 15–85 y	693	36.2	40.1	23.4	Montelukast 10 mg/d	Loratadine 10 mg/d	None	2	DNSS, NSS, CSS, DESS, RQLQ
van Adelsberg 2003b	the US	R, DB	SAR patients aged 15–82 y	628	36.3	33.2	22.4	Montelukast 10 mg/d	Loratadine 10 mg/d	None	4	DNSS, NSS, CSS, DESS, RQLQ
Lee 2004	UK	R, DB, CO	Adult PAR patients	12	42	33.3	NR	Montelukast 10 mg/d	Fexofenadine 180 mg/d	None	1	DNSS
Di Lorenzo 2004	Italy	R, DB	SAR patients aged 12–50 y	40	31.8	35	NR	Montelukast 10 mg/d	cetirizine 10 mg/d	Fluticasone propionate aqueous nasal spray	6	DNSS
Hsieh 2004	China	R, DB	Child PAR patients aged 6–12 y	40	8.1	62.5	NR	Montelukast 5 mg/d	Cetirizine 10 mg/d	None	12	DNSS
Ciebiada 2006-levo	Poland	R, DB, CO	Adult PAR patients aged 18–65 y	20	23.7	30	0	Montelukast 10 mg/d	Levocetirizine 5 mg/d	None	6	DNSS, DESS
Ciebiada 2006-deslo	Poland	R, DB, CO	Adult PAR patients aged 18–65 y	20	34.1	20	0	Montelukast 10 mg/d	Desloratadine 5 mg/d	None	6	DNSS, DESS
Jiang 2006	China	R	PAR patients aged 15–65y	63	28.6	41.3	NR	Zafirlukast 40 mg /d	Loratadine 10 mg/d	None	2	DNSS
Philip 2007	the US	R, DB	PAR patients aged 15–85y	752	35.5	33.1	23.7	Montelukast 10 mg/d	Cetirizine 10 mg/d	None	6	DNSS, RQLQ
Lu 2009-1	the US	R, DB	SAR patients aged 15–85 y	228	35.2	36.3	24.1	Montelukast 10 mg/d	Loratadine 10 mg/d	None	2	DNSS, CSS
Lu 2009-2	the US	R, DB	SAR patients aged 15–85 y	267	30.9	38.9	70.8	Montelukast 10 mg/d	Loratadine 10 mg/d	None	2	DNSS, CSS
Liu 2016	China	R	SAR patients aged 16–69 y	64	37.3	54.2	NR	Montelukast 10 mg/d	Fexofenadine 120 mg/d	Nasal budesonide	4	DNSS, RQLQ
Jia 2017	China	R	PAR patients aged 12–56 y	38	29.7	54.4	0	Montelukast 10 mg/d	Loratadine 10 mg/d	Nasal mometasone	4	DNSS

*LTRAs* leukotriene receptor antagonists, *SAHs* selective H1-antihistamines, *AR* allergic rhinitis, *US* United States, *UK* United Kingdom, *R* randomized, *DB* double blinded, *CO* crossover, *SAR* seasonal allergic rhinitis, *PAR* perennial allergic rhinitis, *NR* not reported, *DNSS* daytime nasal symptoms score, *NSS* nighttime symptoms score, *DESS* daytime eye symptoms score, *CSS* composite symptoms score, *RQLQ* rhinoconjunctivitis quality-of-life questionnaire

### Data quality

Table 2 shows the details of the study quality evaluation. Most of the included RCTs were randomized and double-blind except for three studies, which were randomized but open-label [18, 21, 22]. The methods used for random sequence generation were reported in eight studies and none of the included studies reported the details of allocation concealment. The overall quality score ranged between 2 and 6.

**Table 2:** Details of study quality evaluation via the Cochrane’s Risk of Bias Tool

	Sequence generation	Allocation concealment	Blinding of participants and personnel	Blinding of outcome assessment	Incomplete outcome data	Selective outcome reporting	Other potential threats	Total
Meltzer 2000	Low	Unclear	Low	Low	Low	Low	Unclear	5
Nayak 2002	Low	Unclear	Low	Low	Low	Low	Low	6
Philip 2002	Unclear	Unclear	Low	Low	Low	Low	Unclear	4
van Adelsberg 2003a	Low	Unclear	Low	Low	Low	Low	Unclear	5
van Adelsberg 2003b	Unclear	Unclear	Low	Low	Low	Low	Unclear	4
Lee 2004	Unclear	Unclear	Low	Low	Low	Low	Unclear	4
Di Lorenzo 2004	Unclear	Unclear	Low	Low	Low	Low	Low	5
Hsieh 2004	Low	Unclear	Low	Low	Low	Low	Unclear	5
Ciebiada 2006-levo	Unclear	Unclear	Low	Low	Low	Unclear	Unclear	3
Ciebiada 2006-deslo	Unclear	Unclear	Low	Low	Low	Unclear	Unclear	3
Jiang 2006	Low	Unclear	High	High	Low	Low	Unclear	3
Philip 2007	Low	Unclear	Low	Low	Low	Low	Unclear	5
Lu 2009-1	Low	Unclear	Low	Low	Low	Low	Low	6
Lu 2009-2	Low	Unclear	Low	Low	Low	Low	Low	6
Liu 2016	Unclear	Unclear	High	High	Low	Low	Unclear	2
Jia 2017	Unclear	Unclear	High	High	Low	Low	Unclear	2

### Meta-analysis results

**Fig. 2:**
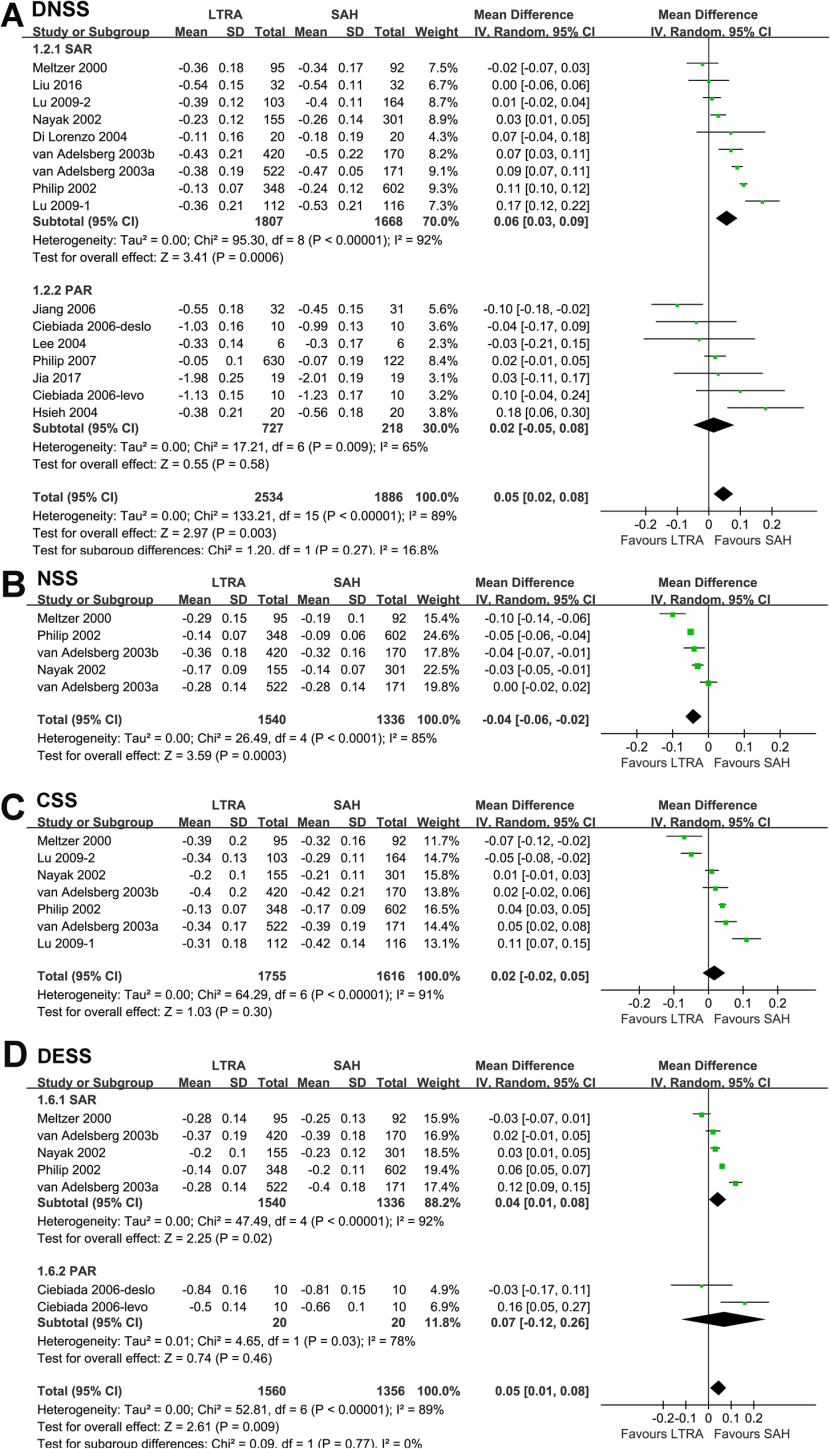
Forest plots for the meta-analysis comparing the effects of LTRAs and SAHs on **A** DNSS, **B** NSS, **C** CSS, and **D** DESS in patients with AR. *LTRAs* leukotriene receptor antagonists, *SAHs* selective H1-antihistamines, *DNSS* daytime eye symptoms score, *NSS* nighttime symptoms score, *CSS* composite symptoms score, *DESS* daytime eye symptoms score

Pooled results with 16 datasets from 14 RCTs showed that treatment with LTRAs was inferior to SAH treatment in terms of the DNSS (MD: 0.05, 95% CI 0.02 to 0.08, *p* = 0.003; Fig. 2A) with significant heterogeneity (*I*^2^ = 89%). Sensitivity analysis by excluding one dataset at a time showed similar results. Subgroup analyses also showed similar results for seasonal AR patients (MD: 0.06, 95% CI 0.03 to 0.09, *p* < 0.001) but not for perennial AR patients (MD: 0.02, CI − 0.05 to 0.08, *p* = 0.58). However, the between-subgroup difference was not statistically significant (*p* = 0.27; Fig. 2A).

Meta-analysis of five studies [9–13] with seasonal AR patients showed that LTRAs were superior to SAHs in terms of the NSS (MD: − 0.04, 95% CI − 0.06 to − 0.02, *p* < 0.001, *I*^2^ = 85%; Fig. 2B). Sensitivity analysis by excluding one dataset at a time showed similar results.

Meta-analysis of seven datasets from six studies [9–13, 20] with seasonal AR patients showed similar CSS between the two treatments (MD: 0.02, 95% CI − 0.02 to 0.05, *p* = 0.30, *I*^2^ = 91%; Fig. 2C). Sensitivity analysis by excluding one dataset at a time also showed similar results.

Pooled results with seven datasets from six RCTs [9–13, 17] showed that treatment with LTRA was inferior to SAH in terms of the DESS (MD: 0.05, 95% CI 0.01 to 0.08, *p* = 0.009, *I*^2^ = 89%; Fig. 2D). Sensitivity analysis by excluding one dataset at a time showed similar results. Subgroup analyses showed similar results for seasonal AR patients (MD: 0.04, 95% CI 0.01 to 0.08, *p* = 0.02) but not for perennial AR patients (MD: 0.07, CI − 0.12 to 0.26, *p* = 0.46). However, the between-subgroup difference was not statistically significant (*p* = 0.77; Fig. 2D).

**Fig. 3:**
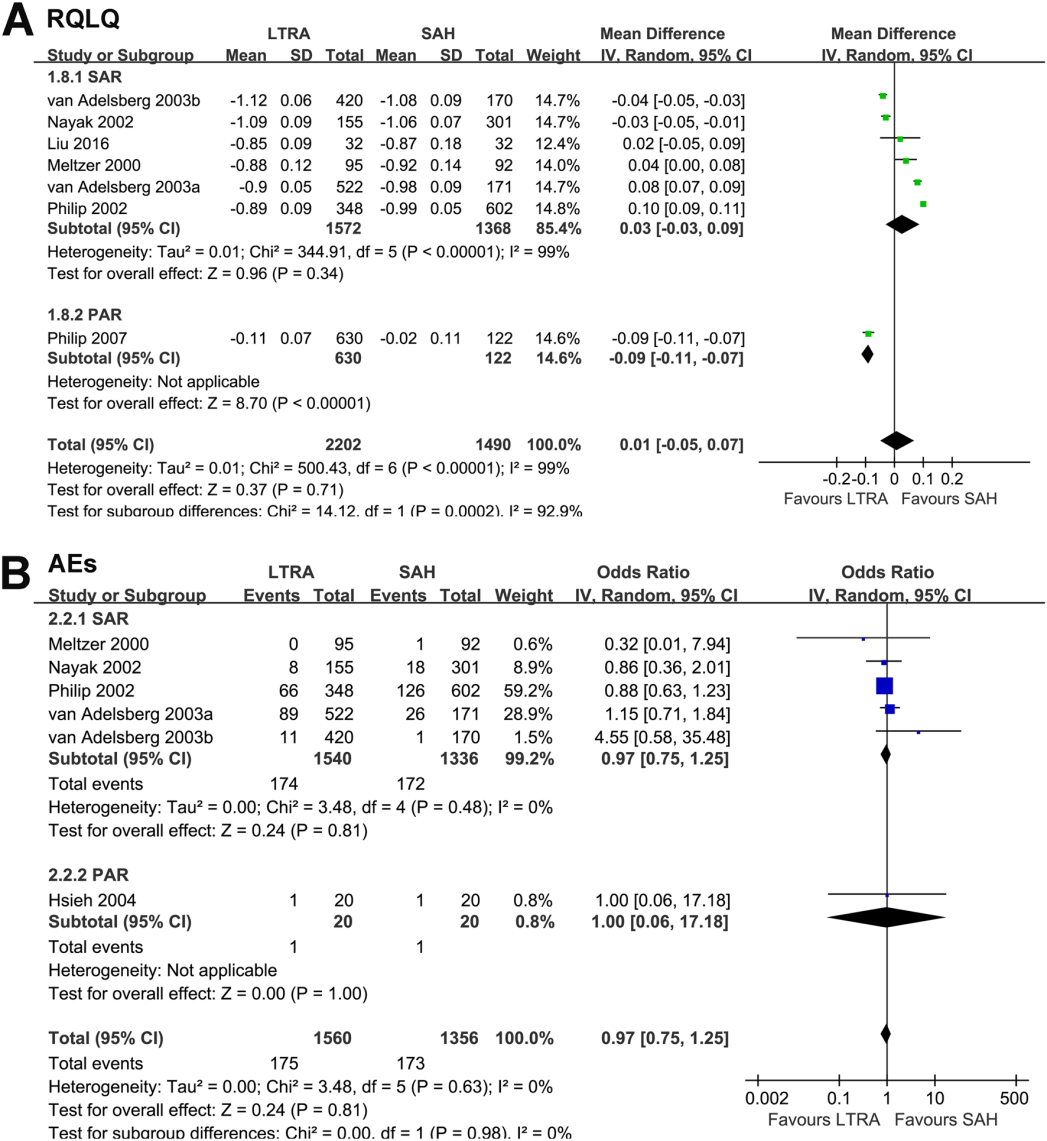
Forest plots for the meta-analysis comparing the effects of LTRAs and SAHs on **A** RQLQ and **B** the incidence of AEs in patients with AR. *LTRAs* leukotriene receptor antagonists, *SAHs* selective H1-antihistamines, *RQLQ* rhinoconjunctivitis quality-of-life questionnaire, *AEs* adverse events

Meta-analysis of seven studies [9–13, 19, 21] showed that RQLQ was not significantly different between the two groups (MD: 0.01, 95% CI − 0.05 to 0.07, *p* = 0.71, *I*^2^ = 99%; Fig. 3A). Sensitivity analysis by excluding one dataset at a time showed similar results. Subgroup analysis showed consistent results for seasonal AR patients (MD: 0.03, 95% CI − 0.04 to 0.09, *p* = 0.34, *I*^2^ = 99%; Fig. 3A). Only one study involving patients with perennial AR showed that LTRAs may be superior to SAHs in terms of the RQLQ (MD: − 0.09, 95% CI − 0.11 to − 0.07, *p* < 0.001; Fig. 3A).

The incidence of AEs was comparable between the groups (six RCTs [9–13, 15], OR: 0.97, 95% CI 0.75 to 1.25, *p* = 0.98, *I*^2^ = 0%; Fig. 3B), which showed similar results in sensitivity analyses and subgroup analyses for seasonal or perennial AR (Fig. 3B).

### Publication bias

**Fig. 4:**
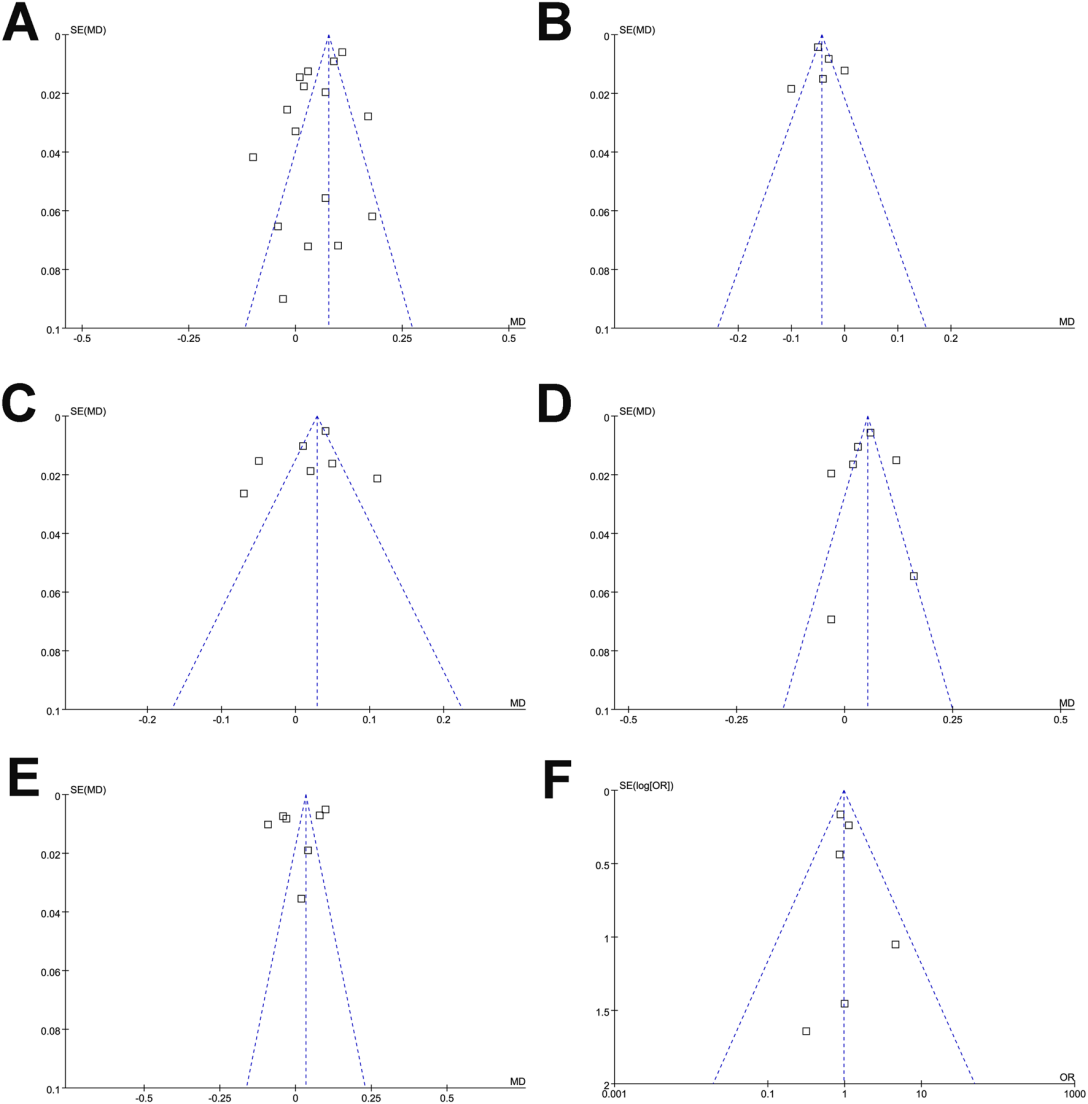
Funnel plots for the meta-analysis comparing the effects of LTRAs and SAHs on **A** DNSS, **B** NSS, **C** CSS, **D** DESS, **E** RQLQ, and **F** the incidence of AEs in patients with AR. *LTRAs* leukotriene receptor antagonists, *SAHs* selective H1-antihistamines, *DNSS* daytime eye symptoms score, *NSS* nighttime symptoms score, *CSS* composite symptoms score, *DESS* daytime eye symptoms score, *RQLQ* rhinoconjunctivitis quality-of-life questionnaire, *AEs* adverse events

The funnel plots were symmetrical, suggesting a low risk of publication bias for the outcomes of the meta-analyses (Fig. 4A–F). Egger’s regression tests showed similar results for the meta-analysis of DNSS (*p* = 0.582). For the other outcomes, Egger’s regression tests were not performed as < 10 datasets were available.

## Discussion

The main findings of the meta-analysis were: (1) LTRAs are inferior to SAHs for improving the daytime nasal symptoms of AR, including stuffy, runny, and itchy nose and sneezing; (2) LTRAs are superior to SAHs for improving the nighttime symptoms of AR, including nasal congestion on awakening, difficulty going to sleep, and nighttime awakenings; (3) the effects of the two medications on the composite symptoms, daytime eye symptoms, and QoL for AR patients are similar; and (4) the incidence of AEs was comparable for patients in both groups. These results suggested that although the two medications were similar in terms of the overall AR symptoms (CSS), eye symptoms (DESS), quality of life (RQLQ), and incidence of AEs, SAHs are more suited for patients with primarily daytime symptoms, while LTRAs are more suited for patients with nighttime symptoms.

A few previous meta-analyses have explored the comparative role of LTRAs and SAHs in the management of AR patients. Xu et al. evaluated nine RCTs published up to 2014 and reported that for seasonal AR patients, LTRAs were inferior to SAHs in terms of the DNSS and CSS, but were superior in terms of the NSS [29]. The authors concluded that SAHs are more appropriate for daytime nasal symptoms while LTRAs are better suited for nighttime symptoms, similar to our findings. However, the superiority of SAHs over LTRAs on CSS suggested that SAHs may be better than LTRAs for improving the overall symptoms of seasonal AR [29]. However, for the CSS outcome, the authors included a dataset with overdosed montelukast (20 mg/d) in a study [9] and another study investigating the acute effects of montelukast [35], which may have confounded the results. Our study, on the other hand, which was limited to head-to-head comparative RCTs with at least 1 week of treatments, showed similar CSS in patients treated with LTRAs and SAHs. The results suggested the two medications had similar efficacy on the overall symptoms of AR, which support their recommendation in the 2017 Japanese Guidelines [25]. Moreover, both the results of our study and Xu et al.’s meta-analyses suggest that LTRAs are better suited for nighttime AR symptoms, which supports the recent recommendation in the 2018 Chinese Guidelines [26]. This is important for clinical practice since the physician’s preference for a certain medication is determined by the main symptoms of the patients. Of note, another meta-analysis published in 2016 aimed to compare the efficacy and safety of SAHs versus montelukast for AR [30]. The results of the meta-analysis showed that montelukast was inferior to SAHs in terms of the DNSS, but superior in terms of the NSS. However, the authors applied a network meta-analysis design and included studies with indirect comparisons between montelukast and SAHs, which also confounded the results [30]. Our study included only direct comparative RCTs and up-to-date evidence and the results provide further confirmation of the comparative efficacy and safety of LTRAs and SAHs in clinical practice. During the preparation of this manuscript, a meta-analysis regarding the role of montelukast as treatment for AR has been published [36]. This study contains a comparative study between montelukast and oral antihistamine for AR. The authors concluded that montelukast was inferior to oral antihistamine in improving DNSS, CSS, DESS, and RQLQ, while montelukast was superior to oral antihistamine in improving NSS [36]. However, regarding antihistamine medication, only studies loratadine were included rather than studies with other SAHs. Besides, no subgroup analysis regarding patients with seasonal or perennial AR was performed. Our study included all available studies comparing LTRAs and SAHs in AR patients, and provided subgroup data regarding the type of AR of the included patients. Accordingly, our meta-analysis could provide a more comprehensive finding regarding the comparative efficacy of LTRAs and SAHs as treatment for AR.

For patients with AR, nighttime symptoms are bothersome, which usually leads to sleep disturbance and daytime tiredness, thereby significantly decreasing QoL in these patients [37]. In a previous study using actigraphy, the author showed that specific sleep disturbances in patients with perennial AR that may result in the increased tiredness, fatigue, and impaired QoL typically experienced in such patients [38]. These facts highlight the importance of our meta-analysis that LTRAs are better suited for nighttime AR symptoms. The potential reasons for the superiority of LTRAs over SAHs on nighttime symptoms in AR patients are unknown. Generally, nasal congestion is considered the main pathological cause of impaired sleep quality in AR patients [39], while nasal congestion may be less relevant to daytime nasal symptoms including stuffy, runny, and itchy nose and sneezing [40]. A previous study indicated that LTRAs are associated with improved nasal congestion [7], which is a late-phase manifestation of increased nasal mucosal inflammation. SAHs are associated with reduced hypersensitivity of the nose and less severe early-phase symptoms during the nasal inflammatory response, such as rhinorrhea, sneezing, and pruritus [8]. Further, LTRAs such as montelukast are usually administered before nighttime [41], which may also be responsible for their superiority in controlling nighttime symptoms. Additional studies are warranted to further explore the potential mechanisms underlying the suitability of the two medications according to the patient’s symptoms.

We performed subgroup analyses to explore the potential differences between LTRAs and SAHs in patients with seasonal or perennial AR. The results of our meta-analysis were mainly driven by studies that included patients with seasonal AR. The differences between LTRAs and SAHs became non-significant when only studies with perennial AR were considered (e.g. DNSS). Therefore, the comparative efficacy and safety of LTRAs and SAHs in patients with perennial AR remain to be clarified in large-scale RCTs. Interestingly, the only study that compared the effects of LTRAs and SAHs on RQLQ in patients with perennial AR showed a superiority of LTRAs over SAHs [19]. The reason for this finding is currently unknown. However, it can be assumed that patients with perennial AR are more likely to have nasal congestion and related sleep disturbance, which may be an important component of poor RQLQ in this population. The superiority of LTRAs over SAHs for nasal congestion and nighttime symptoms may explain the benefits of LTRAs for RQLQ in patients with perennial AR. Unfortunately, the degree of nasal congestion and changes in nighttime symptoms were not evaluated in this study [19]. More clinical studies are needed to validate this hypothesis.

Our study has several limitations. Firstly, the ages of the included patients varied. Due to the lack of study data stratified by ages, we were unable to compare the safety and efficacy of LTRAs and SAHs in pediatric and adult patients. Secondly, significant heterogeneity remained in some outcomes, which may be explained by the differences in patient characteristics, medication regimens, and follow-up durations. Thirdly, LTRAs are suggested to be effective for asthma. LTRAs are assumed to have better efficacy for patients with AR and asthma. Although some of the patients who were included in the studies had asthma, we were unable to compare the efficacy and safety of LTRAs and SAHs in these patients because stratified results were not reported. Finally, in view of the potential preference of LTRAs and SAHs for AR patients according to their symptoms, combined treatment with the two medications may achieve better symptom improvement, which should be validated in future studies.

## Conclusions

The results of this meta-analysis of head-to-head RCTs showed that although both medications are safe and effective in improving the QoL of AR patients, LTRAs are more effective in improving nighttime symptoms but less effective in improving daytime nasal symptoms compared to SAHs. These findings were mainly driven by studies that included seasonal AR patients. Further studies are needed to compare the efficacy and safety of LTRAs and SAHs in patients with perennial AR and to determine the efficacy of a combined treatment with the two medications for AR patients.

## Data Availability

The datasets used and analysed during the current study are available from the corresponding author on reasonable request.

## Abbreviations

AEs: Adverse events
AR: Allergic rhinitis
CIs: Confidence intervals
CSS: Composite symptoms score
DESS: Daytime eye symptoms score
DNSS: Daytime nasal symptoms score
H1R: Histamine 1 receptor
LTRAs: Leukotriene receptor antagonists
MD: Mean difference
NSS: Nighttime symptoms score
QoL: Quality of life
OR: Odds ratio
RCTs: Randomized controlled trials
RQLQ: Rhinoconjunctivitis quality-of-life questionnaire
SAHs: Selective H1-antihistamines
